# Hydrogen-driven asymmetric reduction of hydroxyacetone to (*R*)-1,2-propanediol by *Ralstonia eutropha* transformant expressing alcohol dehydrogenase from *Kluyveromyces lactis*

**DOI:** 10.1186/1475-2859-12-2

**Published:** 2013-01-10

**Authors:** Takahiro Oda, Koji Oda, Hiroaki Yamamoto, Akinobu Matsuyama, Masaharu Ishii, Yasuo Igarashi, Hirofumi Nishihara

**Affiliations:** 1 Department of Bioresource Science, College of Agriculture, Ibaraki University, 3-21-1 Chu-ou, Ami-machi, Inashiki-gun, Ibaraki, 300-0393, Japan; 2 Green Product Development Center, R&D Management, Daicel Corporation, 1-1 Shinko-cho, Myoko, Niigata, 944-8550, Japan; 3 Department of Biotechnology, Graduate School of Agricultural and Life Sciences, The University of Tokyo, 1-1-1 Yayoi, Bunkyo-ku, Tokyo, 113-8657, Japan

**Keywords:** Hydrogen-driven bioconversion, Hydrogen-driven cell factory, NAD-reducing soluble hydrogenase, Alcohol dehydrogenase, Cofactor regeneration, (*R*)-1,2-propanediol, Hydrogen-oxidizing bacterium, *Ralstonia eutropha*

## Abstract

**Background:**

Conversion of industrial processes to more nature-friendly modes is a crucial subject for achieving sustainable development. Utilization of hydrogen-oxidation reactions by hydrogenase as a driving force of bioprocess reaction can be an environmentally ideal method because the reaction creates no pollutants. We expressed NAD-dependent alcohol dehydrogenase from *Kluyveromyces lactis* in a hydrogen-oxidizing bacterium: *Ralstonia eutropha*. This is the first report of hydrogen-driven *in vivo* coupling reaction of the alcohol dehydrogenase and indigenous soluble NAD-reducing hydrogenase. Asymmetric reduction of hydroxyacetone to (*R*)-1,2-propanediol, which is a commercial building block for antibacterial agents, was performed using the transformant as the microbial cell catalyst.

**Results:**

The two enzymes coupled *in vitro* in vials without a marked decrease of reactivity during the 20 hr reaction because of the hydrogenase reaction, which generates no by-product that affects enzymes. Alcohol dehydrogenase was expressed functionally in *R. eutropha* in an activity level equivalent to that of indigenous NAD-reducing hydrogenase under the hydrogenase promoter. The hydrogen-driven *in vivo* coupling reaction proceeded only by the transformant cell without exogenous addition of a cofactor. The decrease of reaction velocity at higher concentration of hydroxyacetone was markedly reduced by application of an *in vivo* coupling system. Production of (*R*)-1,2-propanediol (99.8% e.e.) reached 67.7 g/l in 76 hr with almost a constant rate using a jar fermenter. The reaction velocity under 10% P_H2_ was almost equivalent to that under 100% hydrogen, indicating the availability of crude hydrogen gas from various sources. The *in vivo* coupling system enabled cell-recycling as catalysts.

**Conclusions:**

Asymmetric reduction of hydroxyacetone by a coupling reaction of the two enzymes continued in both *in vitro* and *in vivo* systems in the presence of hydrogen. The *in vivo* reaction system using *R. eutropha* transformant expressing heterologous alcohol dehydrogenase showed advantages for practical usage relative to the *in vitro* coupling system. The results suggest a hopeful perspective of the hydrogen-driven bioprocess as an environmentally outstanding method to achieve industrial green innovation. Hydrogen-oxidizing bacteria can be useful hosts for the development of hydrogen-driven microbial cell factories.

## Background

The importance of bioprocess reactions that work effectively under mild conditions is growing rapidly as increasing demand for green innovation of industrial process. The conversion of a ketone to a corresponding alcohol represents a common redox-reaction in organic chemistry [1,2]. Dehydrogenases and reductases are promising biocatalysts for such reactions, of which the vast majority require nicotinamide cofactors such as NADH and NADPH as reductant. Given the costs of these cofactors, their stoichiometric use is economically infeasible. Therefore, various *in situ* regeneration methods including chemical, photochemical, electrochemical, and enzymatic reactions have been investigated [1-5]. Enzymatic approaches are particularly attractive for industrial process because of their high selectivity and efficiency. For example, a bioreduction system for the production of chiral alcohols has been reported using an *Escherichia coli* transformant, in which NAD(P)H-dependent carbonyl reductase and glucose dehydrogenase were heterologously co-expressed [1]. Continuous production of chiral alcohol was achieved through the *in vivo* coupling reaction of these enzymes, but production of equimolar amounts of a waste product, gluconate, is problematic. Formate dehydrogenase is known as another useful enzyme for cofactor regeneration [3], the benefits of which include its use of formate as an inexpensive substrate for cofactor reduction and generation of gaseous CO_2_ as the only by-product. It does not affect activities of the enzymes and it is easily separated. However, generation of CO_2_ is problematic in global warming issues. The low catalytic activity of formate dehydrogenase is also cited as an important shortcoming.

Hydrogen, a strong, inexpensive reductant, is also innocuous to the enzymes which will be coupled to a hydrogenase reaction for production of objective products. Importantly, oxidation of hydrogen by hydrogenase causes no pollution. Therefore, a hydrogen-driven bioconversion system can present an environmentally ideal method. The NAD-reducing soluble hydrogenase (ReSH) from *Ralstonia eutropha* (formerly *Alcaligenes eutrophus*), which is classified in Group 3 of [NiFe]-hydrogenase [6], is promising for such biocatalytic application as the enzyme is notably oxygen-tolerant [7,8]. Several reports have described that an *in vitro* coupling reaction by ReSH and NAD-dependent dehydrogenases functions effectively [9-11]. Permeabilized cells of *R. eutropha* were also evaluated as whole-cell coenzyme regeneration catalysts in an organic–aqueous two-phase system, in which reduction of cyclohexanone was examined using *in vitro* coupling reaction of a commercial horse-liver alcohol dehydrogenase and permeabilized *R. eutropha* cells [12]. The coupling reaction by two permeabilized cells, *R. eutropha* and *Gluconobacter oxydans*, has also been examined for reduction of 2-octanone to produce (*S*)-2-octanol using H_2_ as reductant [13]. The NADP-reducing hydrogenase I from *Pyrococcus furiosus* has also been coupled with *Thermoanaerobium* sp. alcohol dehydrogenase *in vitro* for reduction of acetophenone and (2*S*)-hydroxy-1-phenylpropanone, which have been converted to corresponding chiral alcohols with total turnover numbers (mol product/mol consumed cofactor) of 100 and 160, respectively [14]. However, the *in vivo* coupling reaction performed in *E. coli* cells [1], which must be more advantageous as a practical system, has not been reported to date.

**Figure 1:**
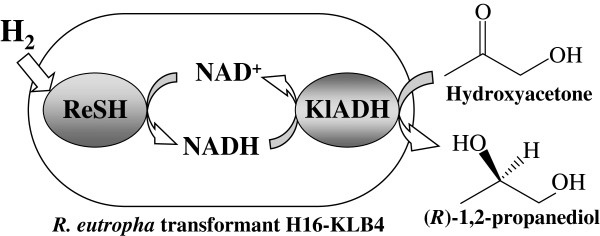
**Hydrogen-driven asymmetric reduction of hydroxyacetone by *R. eutropha *transformant expressing alcohol dehydrogenase from *K. lactis.*** The diagram shows continuous production of (*R*)-1,2-propanediol by *in vivo* coupling reaction of indigenous soluble NAD-reducing hydrogenase (ReSH) and alcohol dehydrogenase from *K. lactis* (KlADH) expressed under the control of ReSH promoter.

In this study, we expressed alcohol dehydrogenase (KlADH) from *Kluyveromyces lactis*[15] in *R. eutropha* cells and evaluated the *in vivo* coupling reaction of ReSH and KlADH (Figure 1). Asymmetric reduction of hydroxyacetone to (*R*)-1,2-propanediol, which is used as a commercial building block for synthesis of antibacterial agents, was examined using the transformant cells as H_2_-driven microbial catalysts in the presence of hydrogen.

## Results and discussion

### H_2_-driven reduction of hydroxyacetone by *in vitro* enzymatic coupling of ReSH and KlADH

To confirm the coupling reaction of ReSH and KlADH *in vitro*, reduction of hydroxyacetone was performed in 1 ml of reaction mixture containing 0.2 U of each enzyme solution and 1 μmol of NADH (1 mM) in vials under H_2_ atmosphere. When 2% (v/v, 294 μmol in the reaction mixture) of hydroxyacetone was subjected for the reaction, 235–252 μmol of the substrate was reduced to 1,2-propanediol in 20 hr. Reduction of 240 μmol of substrate is expected by the 0.2 U of each enzyme activity in 20 hr, indicating that the coupling reaction proceeded effectively without a marked decrease of either ReSH or KlADH activity during the period. This result is attributable to the characteristic of hydrogenase responsible for NADH regeneration, which creates no by-product affecting the enzyme performance. The product was reduced to 134–158 μmol (55.8–65.8% of the expected amount; 240 μmol) when the initial concentration of hydroxyacetone was increased to 3% (v/v).

### Heterologous expression of KlADH in *R. eutropha* H16

We consider that it is important to express KlADH in *R. eutropha* H16 coordinately with indigenous ReSH in a similar level for effective *in vivo* coupling reaction. For this purpose, expression of KlADH was examined by using ReSH promoter. When the transformant H16-KLB4 was cultivated aerobically by hydrogenase derepressing FGN medium for 24 hr, the respective ReSH and KlADH activities in the soluble fraction (SF) were 3.22 U/ml and 4.64 U/ml. The SF prepared from *R. eutropha* H16 cultivated in the same condition showed ReSH activity of 4.10 U/ml and no detectable ADH activity. Consequently, the cell suspension of H16-KLB4 was subjected to H_2_-driven reduction of hydroxyacetone to examine ReSH-KlADH *in vivo* coupling reaction.

### H_2_-driven asymmetric reduction of hydroxyacetone by *R. eutropha* transformant expressing KlADH: *in vivo* coupling reaction of ReSH and KlADH

**Figure 2:**
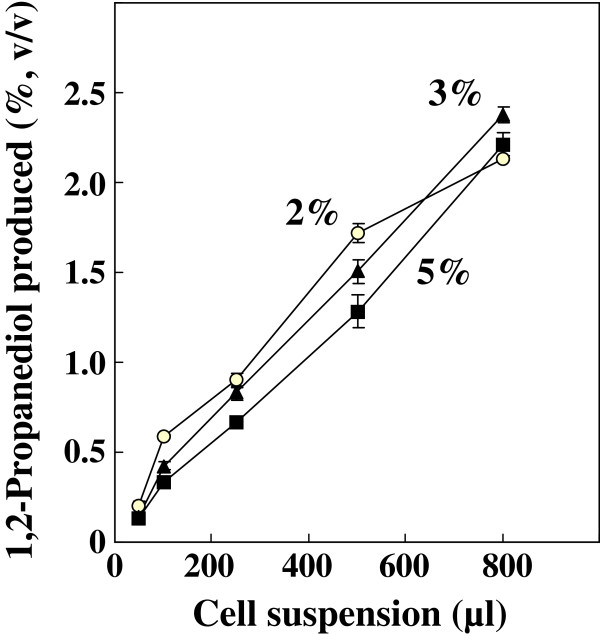
**Hydrogen-driven production of 1,2-propanediol by *R. eutropha *transformant H16-KLB4 using various amount of cell suspension.** The initial concentration of hydroxyacetone (v/v) in the reaction mixture was 2% (○), 3% (*black triangle*), or 5% (*black square*). The SF prepared from the cell suspension (1 g wet cell/5 ml reaction buffer) used for the reaction contained 2.25 U/ml and 1.73 U/ml of ReSH and KlADH activities, respectively**.** In each condition, reaction was performed in two vials.

Reduction of hydroxyacetone proceeded by *in vivo* coupling reaction of ReSH and KlADH in *R. eutropha* H16-KLB4 cells in vials (Figure 2). The SF prepared from the cell suspension used for the reaction contained 2.25 U/ml and 1.73 U/ml of ReSH and KlADH activities, respectively. No NADH was added to the reaction system: only indigenous cofactor was used for the reaction. The production of 1,2-propanediol was increased by increasing the cell suspension used for the reaction. When 800 μl of cell suspension was used, 2% (v/v) of hydroxyacetone was reduced completely (conversion yield; calculated as 99.1%) and the reaction presumably stopped within 20 hr. The decrease of reaction velocity at higher initial concentration of hydroxyacetone was lowered markedly compared to *in vitro* enzymatic coupling reaction. When 2% (v/v, 294 μmol in the reaction mixture) of hydroxyacetone was subjected to the reaction using 500 μl of cell suspension, for example, the concentration of 1,2-propanediol reached 1.72% (v/v, 253 μmol in the reaction mixture) in 20 hr. Relative to this productivity, 88.4% and 74.4% of product were obtained even though the initial concentrations of hydroxyacetone were increased respectively to 3% and 5%. Goldberg et al. [16] reviewed that biocatalytic ketone reduction becomes more stable by using cell catalysts compared to reactions with isolated enzymes in most cases because the enzymes are able to react in their natural environment inside the cell. Our result also showed such advantage of utilizing cell catalyst to stabilize the conversion reaction.

**Figure 3:**
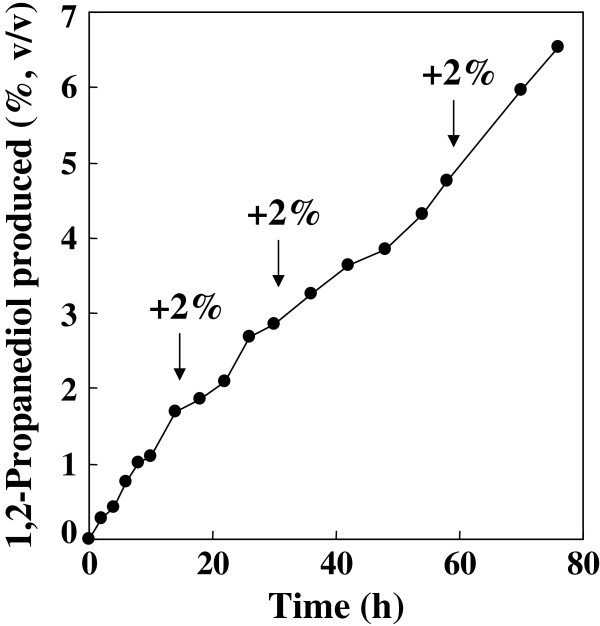
**Hydrogen-driven fed-batch production of 1,2-propanediol by *R. eutropha *transformant H16-KLB4 with a jar fermenter.** The reaction started with 120 ml of reaction mixture containing 60 ml cell suspension (1 g wet cell/5 ml reaction buffer) and 2% (v/v) hydroxyacetone. Hydroxyacetone was added periodically as indicated by arrows as the reaction proceeded. The SF prepared from the cell suspension used for the reaction contained 2.84 U/ml and 1.38 U/ml of ReSH and KlADH activities, respectively.

The fed-batch conversion of hydroxyacetone was investigated further using a jar fermenter under continuous flow of hydrogen (Figure 3). Production of 1,2-propanediol continued for long period with an almost constant rate, which reached 6.54% (v/v, 67.7 g/l) after 76 hr reaction with average reaction velocity of 0.89 g/l/hr. According to this average reaction velocity, 2% (v/v, 294 mmol/l) of substrate periodically added to the reactor was converted completely in every 25.2 hr. The conversion yield of substrate supplied in total (8%, v/v) was calculated as 75.6% in 76 hr reaction. The obtained average reaction velocity corresponds to 28.3% of the expected value (3.15 g/l/hr) calculated from the lower enzyme unit (KlADH) in the SF prepared from a cell suspension used for the reaction (ReSH, 2.84 U/ml; KlADH, 1.38 U/ml). The 76 hr reaction sample showed 99.8% enantiomeric excess of (*R*)-1,2-propanediol.

**Figure 4:**
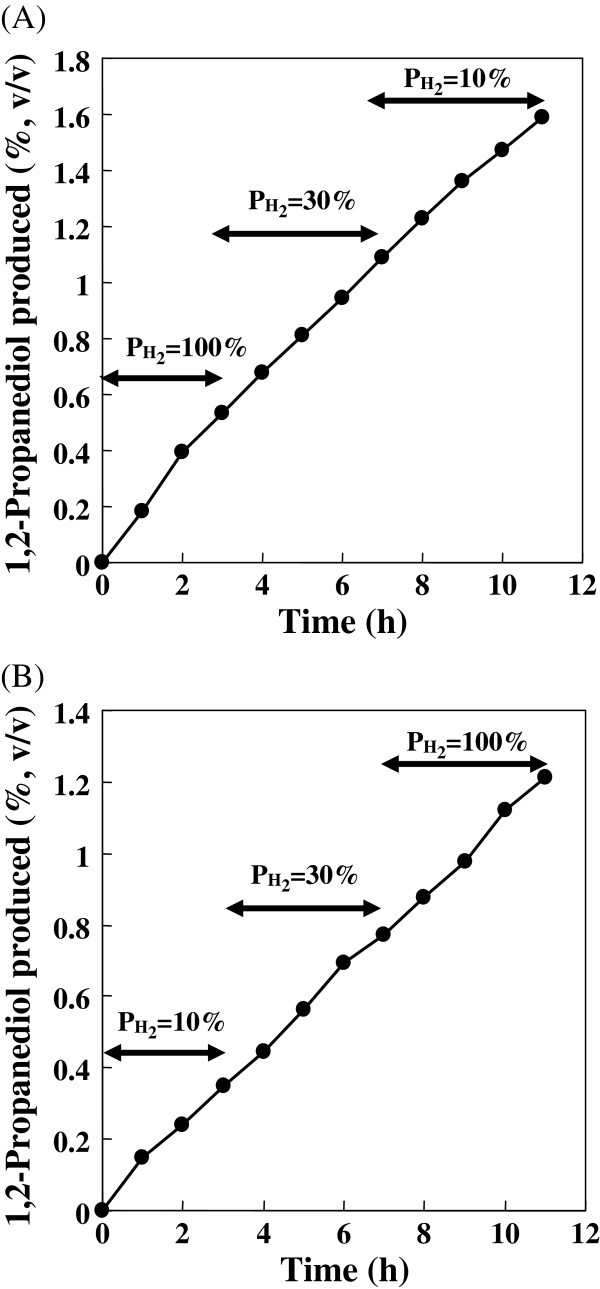
**Influence of P**_**H2 **_**on the H**_**2**_**-driven reaction by *R. eutropha *transformant with a jar fermenter.** The reaction mixture (120 ml) contained 110 ml cell suspension of H16-KLB4 (1 g wet cell/5 ml reaction buffer) and 2% (v/v) hydroxyacetone. (**A**) As the figure shows, the P_H2_ supplied to the reaction mixture decreased from 100% to 10% in a stepwise manner. The SF prepared from the cell suspension used for the reaction contained 1.90 U/ml and 2.85 U/ml of ReSH and KlADH activities, respectively. (**B**) As the figure shows, the P_H2_ supplied to the reaction mixture was increased from 10% to 100%. The SF prepared from the cell suspension used for the reaction contained 1.86 U/ml and 3.61 U/ml of ReSH and KlADH activities, respectively.

**Figure 5:**
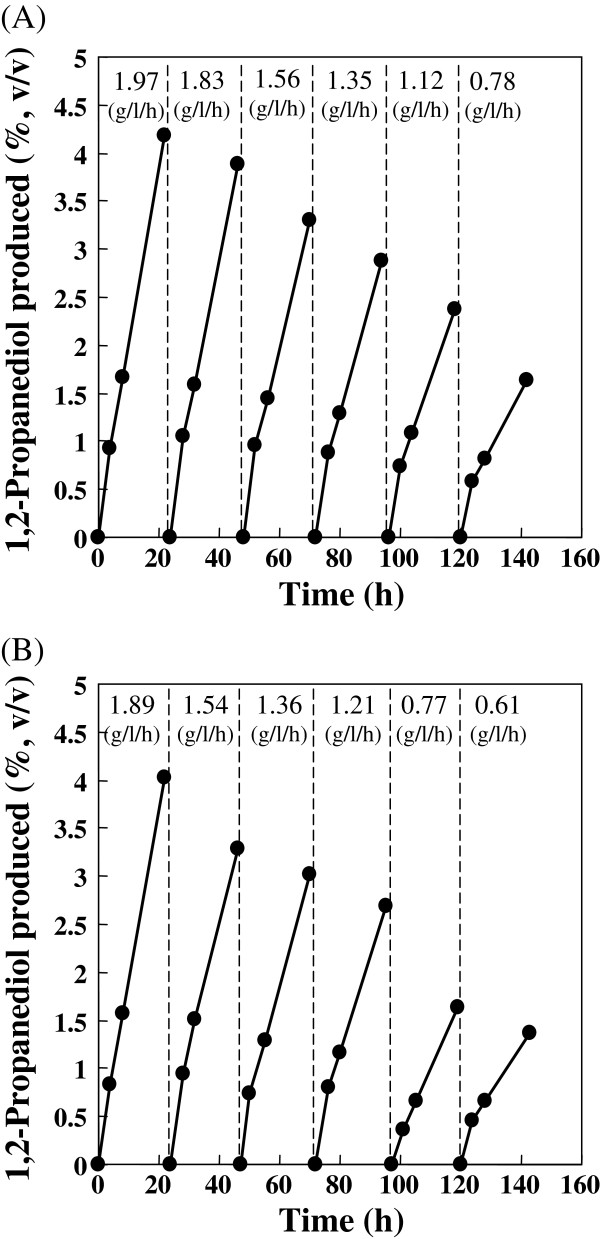
**Repetitive batch reaction by *R. eutropha *transformant H16-KLB4 with a jar fermenter.** The vertical dotted lines represent the beginning of a new batch reaction. The reaction started with 120 ml of reaction mixture containing 110 ml cell suspension (1 g wet cell/5 ml reaction buffer) and 2% (v/v) hydroxyacetone. Hydroxyacetone was additionally supplied to the reaction mixture as the reaction proceeded in each batch reaction. Cells were harvested by centrifugation after 22–23 hr reaction, and were re-suspended in reaction buffer to make 120 ml of reaction mixture for the next batch reaction. The average reaction velocity of the respective batch reaction is shown in the figure. Cell harvest and re-suspension procedures were conducted anaerobically using a vinyl anaerobic chamber (Coy Laboratory Products Inc., MI, USA) (**A**) or were done aerobically under air (**B**). The SF prepared from the cell suspension used for the reaction contained 1.38 U/ml and 1.76 U/ml (**A**) and 1.75 U/ml and 1.62 U/ml (**B**) of ReSH and KlADH activities, respectively.

The reaction velocities varied depending roughly on the ReSH and KlADH activities present in the cells freshly cultivated for each reaction. For example, average reaction velocities of 1.08 g/l/h (2.77 U/ml ReSH and 2.45 U/ml KlADH in the cell suspension) and 0.78 g/l/h (1.15 U/ml ReSH and 1.36 U/ml KlADH in the cell suspension) were obtained in the same working conditions (data not shown). However, reactions continuously proceeded almost linearly in each batch reaction, as also observed in the experiments shown by Figures 4 and 5.

Two reports have described a H_2_-driven coupling reaction using *R. eutropha* cells for cofactor regeneration, but they are *in vitro* coupling systems and performed in an organic–aqueous two-phase system with vials. Andersson et al. [12] combined commercial horse-liver alcohol dehydrogenase and permeabilized *R. eutropha* cells for reduction of cyclohexanone to cyclohexanol. The conversion yield of 200 μmol cyclohexanone in 2 ml heptane (0.2 ml aqueous phase) reached 98% (9.8 g/l product) in the presence of 1 μmol NAD, but the reaction velocity decreased greatly after 24 hr. Rundbäck et al. [13] studied reduction of 2-octane to (*S*)-2-octanol by the combination of permeabilized cells of *Gluconobacter oxydans* and *R. eutropha*. The conversion yield of 200 mM substrate in 0.25 ml *n*-dodecane (1 ml aqueous phase) reached about 75% (ca. 20 g/l) in the presence of 2 mM NAD. In reaction of hydrophobic substances, the use of a second phase of an organic solvent acts as a reservoir delivering substrate to aqueous phase. The organic phase also helps to reduce possible toxicity of substrate and/or product to biocatalysts present in aqueous phase [17]. It is intriguing to consider how the *in vivo* coupling system by transformant cells stabilizes such reactions of hydrophobic substrates using an organic–aqueous two-phase system.

### Influence of P_H2_ on H_2_-driven reaction by *R. eutropha* transformant H16-KLB4

Influence of P_H2_ of the headspace gas to the H_2_-driven reaction was investigated in vials. The reaction mixture (1 ml) contained 500 μl of cell suspension and 3% (v/v) hydroxyacetone. The amounts of 1,2-propanediol produced in 20 hr reaction were almost identical between 100% and 10% P_H2_ (gas mixture of H_2_ and N_2_, 1 atm) (data not shown). The result was confirmed further by reaction with a jar fermenter (Figure 4). The reaction velocity did not change greatly by the P_H2_ value of the introduced gas higher than 10%. In Figure 4A, the reaction was initiated by supplying 100% hydrogen, and the P_H2_ was reduced to 30%; then to 10% in a stepwise manner after several hours in each condition. The average reaction velocity during the respective condition was 1.84 g/l/hr (100% H_2_), 1.35 g/l/hr (30% P_H2_) and 1.24 g/l/hr (10% P_H2_). When gas P_H2_ was increased from 10% to 100% in reverse, 1.21 g/l/hr (10% P_H2_), 1.13 g/l/hr (30% P_H2_) and 1.17 g/l/hr (100% H_2_) average reaction velocities were obtained (Figure 4B). Gas mixtures of P_H2_ of less than 10% could not be prepared because of the range limit of the thermal mass flow meter, but it was confirmed that the presence of 10% H_2_ in gas mixture was sufficient to maintain the reaction velocity equivalent to that by 100% hydrogen. The saturated H_2_ concentration in water phase is 68 μM at 30°C under gas phase (1 atm) containing 10% P_H2_. The result is consistent with the *K*_*m*_ value of ReSH, which is obtained experimentally as 6.1–11.9 μM [18,19].

### Repetitive batch reaction by *R. eutropha* transformant H16-KLB4 with a jar fermenter

Repetitive H_2_-driven reaction by the transformant was investigated using a jar fermenter under continuous flow of hydrogen. The reaction was started with 2% (v/v) of hydroxyacetone, which was added successively to the reaction mixture as the reaction proceeded. Cells were harvested by centrifugation after a batch reaction of 22–23 hr, and were subjected to the next batch reaction (Figure 5). When the procedure of cell harvest and resuspension in a fresh reaction buffer was operated anaerobically to protect ReSH from oxidative inactivation, average reaction velocities were maintained above 1 g/l/hr during five batch reactions. The product reached 189 g/l in all by six batch runs (total net reaction time; 132 hr), in which the average reaction velocity obtained using the sixth batch reaction corresponded to 39.6% of the first batch (Figure 5A). Most hydrogenases are sensitive to oxygen, which causes loss of catalytic activity by oxidation of active site and/or FeS clusters under air atmosphere [20]. However, the cell activity was not greatly affected by handling in air. When cell harvest and resuspension procedures were operated under air, average reaction velocities higher than 1 g/l/hr persisted until the fourth batch reaction. The total 1,2-propanediol produced by six batch reactions reached 166 g/l (total net reaction time; 135 hr), in which 32.3% of average reaction velocity relative to that of the first batch was retained in the sixth batch reaction (Figure 5B). The results are attributable to the high oxygen tolerance of ReSH [7,8]. In addition, there will be an advantage of *in vivo* coupling system in maintaining lower oxygen level inside the cell.

## Conclusions

The coupling reaction of ReSH and KlADH continued in the presence of hydrogen in both *in vitro* and *in vivo* systems. This report of a hydrogen-driven bioconversion by *in vivo* coupling system is the first describing the use of *R. eutropha* transformant expressing KlADH (Figure 1). The *in vivo* coupling reaction proceeded continuously only by cell suspension, which did not require exogenous addition of a cofactor. The concentration of (*R*)-1,2-propanediol reached 67.7 g/l by periodical addition of hydroxyacetone without a marked decrease of reaction velocity. Reuse of transformant cells became available by *in vivo* coupling reaction. Moreover, the presence of 10% P_H2_ retained a reaction velocity equivalent to that by 100% hydrogen, indicating the availability of crude hydrogen gas from various sources. These results suggest a hopeful perspective of hydrogen-driven bioprocesses using *in vivo* coupling system to achieve industrial green innovation.

## Methods

### Bacterial strains, plasmids, and cultivations

Strains and plasmids used for this study are presented in Table 1. *Ralstonia eutropha* H16 was used as a host for expression of KlADH [15]. *E. coli* XL-1 Blue (Stratagene Cloning Systems Inc., Amsterdam, Netherlands) was used as a host in standard cloning procedure, and *E. coli* S17-1 [21] was used in conjugative plasmid transfer. Strains of *R. eutropha* were cultivated heterotrophically in modified LB medium, FN medium, or FGN medium at 30 °C [22]. FN medium (pH 7.0) contained the following components per liter: fructose 4 g; Na_2_HPO_4_ · 12H_2_O 9 g; KH_2_PO_4_ 1.5 g; NH_4_Cl 2 g; MgSO_4_ · 7H_2_O 0.2 g; CaCl_2_ · 2H_2_O 10 mg; FeCl_3_ · 6H_2_O 5 mg; NiCl_2_ · 6H_2_O 19 mg. FGN medium (pH 7.0) contained the following components per liter: fructose 2 g; glycerol 2 ml; Na_2_HPO_4_ · 12H_2_O 9 g; KH_2_PO_4_ 1.5 g; NH_4_Cl 2 g; MgSO_4_ · 7H_2_O 0.2 g; CaCl_2_ · 2H_2_O 10 mg; FeCl_3_ · 6H_2_O 5 mg; NiCl_2_ · 6H_2_O 190 mg. For preparation of hydrogenase derepressed cells, cultivation was done aerobically in FGN medium for 24 hr [22] after pre-cultivation using modified LB medium. Strains of *E. coli* were grown aerobically in LB medium at 37°C [23]. Solid medium contained 1.5% agar (w/v). Antibiotics were supplemented as follows when necessary: 15 μg/ml of tetracycline, 100 μg/ml of ampicillin. *E. coli* HB101 (Takara Bio Inc., Tokyo, Japan) transformed by pSE-KLB2 [15] which possesses KlADH gene in pSE420 (Invitrogen Corp., CA, USA) was used for preparation of the SF containing KlADH. The transformant was cultivated in LB medium containing ampicillin and IPTG was added to the culture to be 0.1 mM when O.D. at 660 nm reached 0.6–0.7. Cells were cultivated another 4 hr and harvested for preparation of the SF.

**Table 1:** Bacterial strains and plasmids used for this study

**Strain or plasmid**	**Relevant characteristic(s)**	**Source or reference**
Strains		
*R. eutropha*		
H16	Wild type	DSM 428, ATCC 17699
H16-KLB4	H16 transformed by pEDY-KLB4	This study
*E. coli*		
XL-1 Blue	*recA1 endA1 gyrA96 thi-1 hsdR17 supE44 relA1 lac* [*F’ proAB lacIq lacZΔM15 Tn10* (*Tc*^*r*^)]	Stratagene Cloning Systems Inc.
S17-1	Tra^+^*recA pro thi hsdR*, *chr*::RP4-2	21
HB101	F^-^, *leuB6* Δ(*gpt*-*proA*)*62 recA13 thi-1 ara-14 lacY1 galK2 xyl-5 mtl-1 rpsL20*(*Str*^+^) *supE44* Δ(*mcrC*-*mrr*)	Takara Bio Inc.
Plasmids		
pBluescript KS (+)	Ap^r^*lac*Z’, T7 gene 10 promoter, f1 *ori*	Stratagene Cloning Systems Inc.
pSE-KLB2	1,183-bp *Eco*RI-*Afl*II fragment containing the complete KlADH gene in pSE420	15
pCH591	260-bp *Hin*dIII-*Nde*I fragment containing the ReSH promoter in Litmus 29	25
pEDY309	RK2 *ori*, Tc^r^, Mob^+^	25
pBlueKLB3F	401-bp PCR fragment containing a part of KlADH gene in *Eco*RV digested pBluescript KS (+)	This study
pCH-KLB2	385-bp *Nde*I-*Xba*I fragment from pBlueKLB3F in pCH591 downstream of ReSH promoter	This study
pCH-KLB3	1,005-bp *Pfl*MI-*Xba*I fragment from pSE-KLB2 in pCH-KLB2	This study
pEDY-KLB4	*Eco*RV-*Sna*BI fragment containing a complete set of the ReSH promoter and KlADH gene from pCH-KLB3 in *Swa*I digested pEDY309	This study

### Construction of KlADH expression vector and transformation of *R. eutropha* H16

Standard DNA techniques were used for DNA manipulation [24]. The plasmid pSE-KLB2 containing the complete 1,155-bp KlADH gene [15] was used as a PCR template to introduce *Nde* I site at the initiation codon ATG and *Xba* I site at the end of the amplified PCR fragment. The following oligonucleotides were used as primers. Therein, designed restriction sites are underlined and mismatched nucleotides to the original sequence are shown in lower case letters: 5^′^-GAATTCTcatATGCGTGCATTAGCTTATTTCGG-3^′^ and 5^′^-AACAGTTcTAGAACCCTCTTTGACAAGC-3^′^. The amplified 401-bp fragment was phosphorylated using a Mighty cloning reagent set (Takara Bio Inc.) and cloned to *Eco* RV site of pBluescript KS (+) (Stratagene Cloning Systems Inc.) to confirm the sequence. A thermosequenase primer cycle sequencing kit (GE Healthcare UK Ltd., Buckinghamshire, England) and DNA sequence LIC-4200S (Li-Cor Inc., Lincoln, NE, USA) were used for sequencing. The PCR fragment of the obtained recombinant plasmid pBlueKLB3F, whose authenticity of the sequence was confirmed, was digested by *Nde* I and *Xba* I and was ligated to the corresponding site of the pCH 591 [25]. The resulting plasmid pCH-KLB2 contained the initial 385-bp fragment of KlADH gene at the downstream of ReSH promoter. The plasmid was digested by *Pfl* MI and *Xba* I, into which the 1,005-bp *Pfl* MI-*Xba* I fragment from pSE-KLB2 was introduced. The resulting plasmid pCH-KLB3 contained a complete set of the ReSH promoter and KlADH gene. The *Eco* RV-*Sna* BI fragment from pCH-KLB3 containing the set was introduced to *Swa* I site of pEDY 309 [25]. The resulting plasmid pEDY-KLB4 was transformed into *E. coli* S17-1 and transferred to *R. eutropha* H16 by spot mating, by which *R. eutropha* transformant H16-KLB4 was obtained. Purification of the PCR amplified fragment and extraction of DNA fragment from the gel strip were done using gel extraction kit (QIA quick; Qiagen GmbH, Hilden, Germany). The plasmids pCH591 and pEDY309 were kindly donated by Dr. B. Friedrich and Dr. O. Lenz (Humboldt Universität zu Berlin, Germany).

### Preparation of SF

The cells were washed using 50 mM potassium phosphate (KP) buffer (pH 7.0) and centrifuged at 7,000 × *g* for 20 min at 4°C. The washed cells were suspended homogeneously in the same buffer to be 1 g wet cell/5 ml and were broken twice (50 W, 5 min, Sonifier 250; Branson Ultrasonics Corp., CT, USA). Cell debris and unbroken cells were removed by centrifugation at 7,000 × *g* for 30 min at 4°C. Membranes in the cell-free extract were removed by ultracentrifugation at 100,000 × *g* for 1 hr at 4°C. The supernatant was used as SF.

### Enzyme assays

NAD-reducing hydrogenase activity was determined spectrophotometrically at 30°C by following the H_2_-dependent reduction of NAD as an electron acceptor using glass cuvettes sealed with a rubber stopper and an aluminum cap [26]. The assay mixture contained 2 mM NAD in 50 mM KP buffer (pH 7.0). NAD-reducing activity of KlADH was similarly measured spectrophotometrically at 30°C in the presence of 20 mM (*R*)-1,2-propanediol and 2.5 mM NAD in 50 mM KP buffer (pH 7.0). One unit of activity was defined as the amount of enzyme that reduced 1 μmol of NAD in a minute. The amount of reduced NAD was determined by the increase of absorbance at 340 nm (ε_340 nm_ = 6.22 mM^-1^ cm^-1^). Protein concentrations were estimated routinely [27] using established procedures of Bio-Rad Protein Assay (Bio-Rad Laboratories Inc., CA, USA).

### H_2_-driven reduction of hydroxyacetone by *in vitro* enzymatic coupling of ReSH and KlADH

SFs containing ReSH and KlADH were prepared respectively from *R. eutropha* H16 and *E. coli* HB101 possessing pSE-KLB2 cells as described above. They were added to give 0.2 U/ml of each enzyme to the reaction mixture containing 1 mM NAD and 2% or 3% (v/v) of hydroxyacetone in 50 mM KP buffer (pH 7.0). The reaction was performed in a 10 ml vial containing 1 ml of reaction mixture, which was sealed with a rubber stopper and an aluminum cap, at 30°C for 20 hr under shaking at 130 rpm. The reaction was initiated by adding hydroxyacetone with a syringe after hydrogen was filled into the vial head space.

### H_2_-driven reduction of hydroxyacetone by *R. eutropha* transformant expressing KlADH: *in vivo* coupling reaction of ReSH and KlADH

The cell suspension of *R. eutropha* H16-KLB4, which was cultivated under hydrogenase derepressing condition, was prepared to be 1 g of wet cell per 5 ml reaction buffer (50 mM KP buffer, pH 7.0) and subjected as microbial catalyst. ReSH and KlADH activities present in the cell suspension were measured by preparing SF as described above. The reaction with 10-ml vials was done as *in vitro* enzymatic coupling reaction under hydrogen atmosphere at 30°C for 20 hr. Gas mixtures of H_2_ and N_2_ (1 atm) were prepared using thermal mass flow meters (Emerson Japan, Ltd., Tokyo, Japan) when the influence of headspace P_H2_ on the reaction was investigated. The reaction mixture (1 ml) contained 50–800 μl cell suspension and 2–5% (v/v) of hydroxyacetone. The reaction with a 250 ml jar fermenter (BMJ-25; Able & Biott Co., Ltd., Tokyo, Japan) was performed at 30°C under agitation at 1,000 rpm. Hydrogen gas was supplied to the reaction mixture with a thermal mass flow meter (Emerson Japan, Ltd.) at a flow rate of 250 ml/min. Gas mixtures of H_2_ and N_2_ (1 atm) were supplied at a total flow rate of 250 ml/min when influence of P_H2_ was investigated. The reaction mixture (120 ml) contained 60 ml or 110 ml cell suspension and 2% (v/v) of hydroxyacetone at the beginning of reaction. When the reaction was continued to reach higher product concentration, hydroxyacetone (2%, v/v) was added periodically to the reaction mixture as the reaction proceeded. The reaction pH was controlled using a pH controller (FC-2000; Tokyo Rikakikai Co., Ltd., Tokyo, Japan).

### Measurement of 1,2-propanediol and chirality analysis

The reaction mixtures of *in vitro* enzymatic reaction were taken to sampling tubes, which were boiled for 5 min to stop the reaction and centrifuged. The supernatants were subjected to quantification of 1,2-propanediol using a gas chromatograph (GC-14B; Shimadzu Corp., Kyoto, Japan) equipped with a column (2.1 m × 3.2 mm internal diameter) packed with 5% Thermon-3000 (Chromosorb W 80/100 AW-DMCS; Shimadzu GLC Ltd., Tokyo, Japan) and a flame ionization detector. The column temperature was 150°C. The samples of *in vivo* coupling reaction were centrifuged to remove *R. eutropha* H16-KLB4 cells, and the supernatants were boiled for 5 min and centrifuged again. The supernatants were subjected to gas chromatography as described above. Optical purity of 1,2-propanediol produced was analyzed using a high performance liquid chromatograph (L-6320; Hitachi, Ltd., Tokyo, Japan) equipped with an OD-H column (25 cm × 4.6 mm internal diameter, Chiralcel; Daicel Corp., Tokyo, Japan) at 40°C. Solvent consisting of *n*-hexane:2-propanol (4:1) was used as mobile phase at a flow rate of 1.0 ml/min. Detection was done at 254 nm. The sample prepared for GC analysis containing ca. 25 μl of 1,2-propanediol was taken to a test tube and dissolved in ethyl acetate after evaporation of the water phase. The solvent was evaporated after filtration with a 0.45 μm filter (Millex LH; Merck KG, Darmstadt, Germany). Then the remaining sample was dissolved in 25 μl of phenylisocyanate and was left to stand for 30 min at 50°C. Ethanol (25 μl) was added to the sample and incubated for 10 min at 50°C. Solvent of the sample was exchanged to the HPLC solvent by evaporation, and was subsequently subjected to HPLC after filtration (Millex LH; Merck KG).

## Competing interests

The authors declare that they have no competing interests.

## Authors’ contributions

HN designed research with the help of HY, AM (gene manipulation and activity measurement of KlADH, GC and HPLC analysis of the product), MI, YI (hydrogen-driven reactions). TO carried out genetic experiments. TO and KO performed hydrogen-driven reactions and analytical experiments. HN supervised experiment and wrote the paper. All authors contributed to improve the manuscript. The final version of the manuscript has been approved by all authors.
